# 

**Published:** 1992-12

**Authors:** 


South West Urological Oncology Group

### WEST OF ENGLAND MEDICAL JOURNAL

**DECEMBER 1992** **Volume 107(iii)**

Copyright © West of England Medical Journal Ltd. 1992

**CONTENTS**

66 **EDITORIALS**
P. Goddard and J. Kabala

67 **Current Trends in the Management of Invasive Bladder Cancer**
G N A Sibley and J Kabala

70 **Current Trends in the Management of Localised Prostate Cancer**
G N A Sibley and J Kabala

73 **New Concepts in the Investigation and Treatment of Prostatic Hyperplasia**
J McLoughlin

77 **The Role of Ultrasound in the Management of Scrotal Disorders**
J McLoughlin

79 **Incontinence in the Elderly**
G W Tobin

**PROCEEDINGS**

82 **South West Urological Oncology Group Autumn Meeting 1991**

84 **South West Surgical Club Autumn Meeting 1992**

86 **Wessex Branch of the Association of Clinical Pathologists Winter Meeting 1992**

**ORIGINAL PAPERS**

90 **Extra-Paramidal Side Effects Associated with Paroxetine**
S D Nicholson

91 **Neuroborreliosis in South Wales**
K K T Lim, B McLean and C J Burns-Cox

92 **Occupational Therapy — Does the Doctor Know?**
E. Bawn

95 **OBITUARIES**
Dr R P Warin
Dr D J Bennett
Professor J H Peacock

